# 

**Published:** 1857-10-01

**Authors:** 


### INDEX TO VOL. X.

A.

Act for regulation of lunatics and lunatic asylums in Scotland, 806
Albinoism, 372
American institutions for the insane, 262
Animals, Mammalian class, their instincts, 144
——— natural modifications and degenerations of, difference between them, 163, 164
Apparitions, 293–302
——— induced by vivid imaginations, 304
Arlidge, John T., M.B., on the asylums of Italy, Germany, and France, 521, 758
Asylums, Annual Meeting of the Medical Officers of, 776
——— in Italy, Germany, and France, 251, 758
——— religious teachers in, 244
——— in Scotland, Act for regulation of, 806
Attendants in asylums, 285
Author-teachers, 78
Autobiography of the insane, 39

B.

Bacon's Philosophy, 37, 82
Belgium, asylums in, 50, 57, 58, 209, 240
——— ——— their former state, 59
——— ——— medical officers of, 243.
Belgium, classification of lunatics in, 52
——— proposed society for protection and cure of invalids in, 819
Bemiss, S. M., M.D., on marriages of consanguinity, 368
Belief, the ultimate fact in psychology, 570
Bethlem Hospital, 379, 495, 551, 588
Blood, effects of mental labour on the, 88
Boismont, M. Brierre de, on the insanity of early life, 622
Brain, condition of, at birth, 640
Brain disease, neglected, 413
——— fever, in children, 630
Bruges, asylums at, 60
Bucks County Asylum and Mr. Millar, 157

C.

Cause, Spinoza's definition of, 323
Capital punishment, in civilized countries, 253
——— ——— its effects on the lower classes, 249
——— ——— statistics of, 403
——— ——— scripturally considered, 247, 252, 259, 260
Children, insanity of, 262
Chloroform, in cases of delirium tremens, 134
——— its use in puerperal insanity, 123
Cities, effect of, on the human race, 203
Civilization and insanity, 338
Commissioner in Lunacy, 412, 806
Consanguinity, marriages of, 368, 374
Consciousness, intellectual, 358
"Conseil de Famille," 51
County asylums, how considered by the public, 549
Cretinism and idiocy, 705
Criminal lunatics, classification of, 647
——— ——— in Ireland, 728
——— ——— treatment of, 553
Criminals, classification of, 660
——— young, effects of punishment of, 655

D.

Dartmoor Prison, insanity at, 409
Deaf and dumb, their notions in written language, 361
Deafness congenital in marriages of consanguinity, 372
Deceit, effects of on the insane, 582
Delirium tremens, chloroform in, 134
Denham, Rev. J. F., on capital punishment, 247
Descartes and Spinoza, their systems, 334
Devon County Lunatic Asylum, 236
Diet of lunatics, 242

Double-consciousness, 308
Dreams, causes of, 297
——— characteristics of, 295
——— different phases of, 297
——— physiological and psychological phenomena of, 293, 297
Dunn, Robert, on physiological psychology, 135, 358

E.

Earle, S. Pliny, on American institutions for the insane, 262
England, amelioration of the insane in, 354
——— lunacy in, 581
Ergotism, 181

F.

Ferrier, Professor, his New Scottish Philosophy, 28
Food, refusal of, by the insane, 788
France, asylums in, 758

G.

George III., his insanity, 95, 355
——— physicians in his case, 98, 110, 112, 117, 120
Gheel, insane colony of, 50, 55, 210, 344, 353
Ghent, asylums at, 68

H.

Hamilton, Sir W., considered as a philosopher, 29, 34
Hanwell asylum, report of, 591
Hawkes, John, on the increase of insanity, 508
Hereditary affections, 187, 504
Hereditary influence, animal and human, 385
Homicidal mania, frequent instances of, 424
Hood, W. Charles, M.D., on the pathology of insanity, 379
——— on the statistics of insanity, 495
Hoppus, Professor, on the psychology of Spinoza, 313
——— on the psychology of Wolf, 735
Human race, degeneracy of the, 159, 202
——— varieties of the, 162
Human knowledge, datum for, 565
Hume, Dr., death of, 412
Hypothesis and research, their value in medicine, 82
Hysterics, forerunner of insanity, 633

I.

Idiocy and cretinism, 705
Idiots, education of, 404
Infanticide, to what attributable, 190
Insane, American institutions for the, 262
——— autobiography of the, 39
——— enactments relating to the, 617
——— incurable, 268
——— isolation of the, 356
——— modern treatment of the, 340, 344
——— their association with sane, 237
——— prolonged shower bath in the treatment of the, 1
——— treatment of the, 621, 788
——— ——— in Germany, 345
Insanity and civilization, 338
——— at the period of puberty, 626
——— circular, 632
——— in early life, 622
——— incipient, symptoms of, 415, 422
——— increase of, 508
——— of George III., by Dr. Ray, 95
——— pathology of, 379
——— preventive cure of, 637
Intellectual consciousness, 358
Ireland, state of lunacy in, 723
Isolation of the insane, 355
Italy, asylums in, 758

J.

Joyous emotions, 151

L.

Language, importance of, 366
Laycock, Thomas, M.D., on the principles and method of observation and research, 81
Legislative enactments, their effects on the psychologist and the insane, 617
Leibnitz, his system of philosophy, 334
Lélut, L. F., on the demon of Socrates, 454
Lewes, G.H., on Philosophical Progress, 671
Lunacy in England and Wales, state of, 581, 802
——— in Ireland, 723
——— in Scotland, 472, 561, 806
Lunatic asylums, Belgian, 50, 209
Lunatics, classification of, 346
——— ——— in Belgium, 52
——— colonization of, 50, 152, 350, 354
——— employment of in the open air, 236, 550

Lunatics, religious denominations of, 518
——— statistics of, in Belgium, 55
——— their diet, 242
——— treatment of in France, 349

M.

Mammalian animals, their instinct, 144
Man, his powers, 159
Mania, homicidal, 424
Mania, incipient, importance of early treatment of, 415, 453
Marriages of consanguinity, 368
——————— effects of, how modified, 374
——————— historical instances of, 375
——————— Roman law as to, 369
Mayo, A. F., Esq., on the compensatory relations between the faculties of order and memory, 715
Mayo, Dr. Thomas, 412
Medical officers, their remuneration, 243
————— of asylums, annual meeting of the, 611, 776
————— in Belgium, 243
Medicine, philosophical, 81
——— scientific, 81
Mental labour, its effects on the blood, 88
Middle classes, insanity in the, 510
Milbank Prison, insanity at, 409
Miller, Mr. Hugh, death of, 415
Millar, Mr., and the Bucks County Asylum, 157
Moral liberty, 638
Morel, Dr. B. A., on the Degeneracy of the Human Race, 159
Morris, the late Jacob G., of Pennsylvania, 273
Murderers, disposal of, 260

N.

Nervous shocks, effects of on females, 310
Non-restraint system in England, 354

O.

Observation and research, lectures on the principles and methods of, by Thomas Laycock, M.D., 81
Opium, its effect on the human race, 175, 191
——— its use in puerperal insanity, 127
Order and memory, compensatory relations between the faculties of, 715

P.

Paralysis, general observations on, by M. Baillarger, 699
Parigot, Dr. F., on civilization and insanity, 338
Pascal, opinion of, by M. Lélut, 466
Pellagra, different modes of treating, 199
Pentonville Prison, insanity at, 409
Philosophers, Scotch, 28
Philosophical medicines, 78
————— progress, 671
Physicians, College of, President, 412
Physiological phrenology, its founder, 156
————— psychology, 135, 358
Plunge baths, use of in insanity, 27
Potato, its effects on the human race, 197
"Principles," importance of in medical science, 8
Prison insanity, foreign, 696
Private asylums in Belgium, 57
Psychiatry, its special study recommended, 354
Psychological literature, foreign, 695
——————— popular, 548
Psychologists, status of, 612, 615
Psychologist, the mission of the, 611
Psychology of Spinoza, 313, 320
————— physiological, 135, 358
Puerperal insanity, statistics of, 125
——————— symptoms of, 125
——————— use of chloroform in, 123
——————— opium in, 127

R.

Ray, Dr., on the insanity of George III., 95
Realism, hypothetic, considered, 578
Reasoning, definition of, 681
Report (Eleventh) of Commissioners in Lunacy, 802

S.

Sardinian Law of Lunacy, 817
Scepticism, rational and irrational, difference between, 678
Scientific medicine, 81
Scotch philosophers, 28
Scotland, lunacy in, 472, 561
——— new Act for regulation of lunatics and asylums in, 806
Scottish Philosophy, old and new, by Professor Ferrier, 28
Scrofula in marriages of consanguinity, 371
Seifert, Dr. Constantine, 412

Sensationalism, 679
Shower baths, prolonged, considered, 1
Silent system of punishment, 658, 664
Socrates, the demon of, 454
Soul, Spinoza's definition of the, 332
Speech, faculty of, where seated, 363, 365
Spencer, Herbert, on the Principles of Psychology, 564
Spinoza an Acosmist, 337
——— and Descartes, their systems, different estimates of, 334
——— his posthumous works, 218
——— his works, 317
——— his moral philosophy, 328
——— his occupations and amusements, 317
——— psychology of, 313
Snape, C., letter to the visitors of the Surrey County Asylum, 1
——— Mr. C., prosecution of, 1
Spirit-rapping, &c., 516
Statistics of insanity, by W. Charles Hood, M.D., 495
Substance, Spencer's definition of, 323
Suicide, by Forbes Winslow, M.D., 413
Suicide, recent instances of, 424
Surrey County Lunatic Asylum, shower bath at, 1
Swedenborg, his visions, 306
Symptoms of disease considered, 83

T.

Tartrate of antimony and bath, use of, in cases of insanity, 12
Thompson, Theophilus, M.D., on the effects of mental labour on the blood, 88
Tobacco, its effect on the human race, 176
Tuke, Dr. Harrington, on the treatment of the insane when food is refused, 788

V.

Vice, cure of, 653, 662
Vital energy, degeneracy of, 414

W.

Waters, A. T. H., on the use of chloroform in puerperal insanity, 123
Webster, John, M.D., on Belgian lunatic asylums, 50, 209, 240
Winslow, Dr. Forbes, his address to the medical officers of asylums, 611
Winslow, Dr. Forbes, his treatment of the insane, 355
Winslow, Forbes, M.D., on suicide, 413
Wolf, psychology of, by Professor Hoppus, 735.

END OF VOL. X.

